# Guillain-Barré syndrome after surgery: a literature review

**DOI:** 10.3389/fneur.2024.1368706

**Published:** 2024-04-04

**Authors:** Xiaowen Li, Chao Zhang

**Affiliations:** Department of Neurology and Tianjin Neurological Institute, Tianjin Medical University General Hospital, Tianjin, China

**Keywords:** Guillain-Barré syndrome, surgery, postoperative complication, retrospective study, management

## Abstract

Guillain-Barré syndrome (GBS) is a rare postoperative complication that is sometimes characterized by serious motor weakness and prolonged weaning from mechanical ventilation. Although the exact nature of the relationship between GBS and the surgical procedure is still unclear, there is a clear increased incidence of GBS in post-surgical patients compared to non-surgical patients. GBS after surgery is unique in several ways. The course of post-surgical GBS unfolds more rapidly than in other situations where GBS develops, the condition is often more severe, and respiratory muscles are more commonly involved. Prompt diagnosis and appropriate treatment are essential, and the condition can worsen if treated inappropriately. Postoperative sedation, intubation, and restraint use make the diagnosis of GBS difficult, as the onset of symptoms of weakness or numbness in those contexts are not obvious. GBS is often misdiagnosed, being attributed to other postoperative complications, and subsequently mishandled. The lack of relevant information further obscures the clinical picture. We sought to better understand post-surgical GBS by performing an analysis of the relevant literature, focusing on clearly documenting the clinical characteristics, diagnosis, and management of GBS that emerges following surgery. We underscore the importance of physicians being aware of the possibility of GBS after major surgery and of performing a variety of laboratory clinical investigations early on in suspected cases.

## Introduction

Guillain-Barré syndrome (GBS) is the most common acute paralytic neuropathy worldwide; it presents primarily as symmetric ascending motor weakness (1, 2). Most often in GBS, the onset of weakness is preceded by a bacterial or viral infection, leading to stimulation of the immune system. Among the antecedent factors that have been associated with GBS, the bacterium *Campylobacter jejuni* is the most common infectious agent associated with GBS (3, 4). Following infection, the acute progression of limb weakness peaks within 4 weeks; 25% of patients may develop respiratory insufficiency and autonomic disturbances. Even with timely and adequate treatment, up to 20% of GBS patients remain unable to walk, and up to 5% of them will die sooner than their healthy cohort (5).

Although GBS is a typical post-infection illness, in most patients with post-surgical GBS, no viral or bacterial infection is mentioned in their medical history, and the only clearly identified antecedent event is the operation. Several reviews and case reports (1, 6–12) suggest the surgical procedure itself is the triggering event for GBS. However, the evidence supporting this notion is meager. Although the exact mechanism underlying post-surgical GBS remains unclear, it is believed that having surgery significantly increases the risk for GBS (6–8). Surgeons may be unfamiliar with post-surgical GBS, as its incidence is low. Thus, they may attribute any weakness or numbness in limbs after surgery to other postoperative complications, leading to the patient being misdiagnosed and treated incorrectly.

In this review of the literature, we describe the clinical characteristics, diagnosis, and management of this syndrome with an emphasis on these factors as they relate to patients that acquired GBS after surgery. Table 1 summarizes the relevant articles on which this review is based.

**Table 1:** Clinical characteristics of 34 cases published within the past two decades (2002–2022) that developed Guillain-Barré syndrome after surgery.

Case no.	First author/y	Sex/age (y)	Type of surgery	Onset of GBS	Initial symptoms	Tendon reflexes in weak limbs	Sensory involvement	Cranial nerve involvement	Autonomic dysfunction	Ventilator support	Serum anti-gangliosid-e antibody	EMG	AD in CSF	Treatment	Other possible triggers	Maximum grade/final grade^a^	Follow-up
1	Yu, 2022 (13)	M/87	Spine Surgery	POD 14	Weakness and paresthesia of legs	Areflexia	Hypoesthesia in limbs	No	No	Yes	Missing^b^	Axonal form	Yes	Supportive care	No	G6/G6	3 y
2	Selcuk, 2022 (14)	F/71	mitral valve replacement	POD 1	Weakness of limbs	Areflexia	Hypoesthesia in limbs	No	No	Yes	Missing^b^	Axonal form	Yes	PE, IVIg	No	G6/G6	25 d
3	Najjari, 2021 (15)	F/32	Gastrogastrost-omy	POD 1	Weakness of limbs	Areflexia	Paresthesia in limbs	No	No	No	Missing^b^	Demyelinating form	Missing^b^	IVIg	frequent vomiting	G3/G0	3 y
4	Chen, 2021 (16)	M/58	Femoral reconstruction	POD 6	Weakness of limbs	Areflexia	Hypoesthesia in limbs	No	No	Yes	Missing^b^	Inexcitable	Yes	PE, IVIg	No	G5/G4	5 y
5	Raut, 2019 (17)	M/32	Cardiac surgery	POD 2	Weakness of limbs	Areflexia	Hypoesthesia in limbs	IX, X	No	Yes	Missing^b^	Demyelinating form	Missing^b^	PE	viral respiratory infection	G5/G4	21 d
6	Aldag, 2017 (18)	M/50	Coronary artery bypass surgery	POD 5	Weakness and paresthesia of legs	Areflexia	Paresthesia in limbs	III, IX, X	Cardio-pulmonary arrest	Yes	Missing^b^	MFS	Normal	PE, IVIg	No	G6/G6	9 d
7	Chen, 2017 (19)	M/57	Elective spine surgery	POD 7	autonomic dysfunction (atrial fibrillation)	Areflexia	Paresthesia in limbs	VII	Atrial fibrillation	Yes	Missing^b^	Demyelinating form	Missing^b^	IVIg, steroids	No	G5/G2	16 mo
8	Li, 2017 (20)	F/29	Cesarean section	POD 8	Weakness of limbs	Areflexia	No	III, IV, VI	No	No	GM1, GD1b	Axonal form	Yes	IVIg	No	G4/G3	1 mo
9	Li, 2017 (20)	F/53	Rathke cyst resection	POD 12	Weakness of limbs	Areflexia	No	No	No	No	Missing^b^	Axonal form	Yes	IVIg	No	G4/G3	1 mo
10	Sahai, 2017 (21)	M/52	Elective spine surgery	POD 17	Weakness of limbs	Areflexia	No	No	No	No	Missing^b^	Missing^b^	Yes	IVIg	No	G4/G3	3 mo
11	Rashid, 2017 (22)	F/62	Revision lumbar surgery	POD 10	Weakness of legs	Areflexia	Numbness in limbs	No	No	Yes	Missing^b^	Axonal form	Missing^b^	IVIg	No	G4/G1	12 mo
12	Samieirad, 2016 (23)	F/39	Internal fixation of mandibular body	POD 4	Weakness of limbs	Hyporeflexia	No	No	Gastric pain	Yes	Missing^b^	Demyelinating form	Missing^b^	Supportive care	No	G5/G0	6 mo
13	Huang, 2015 (11)	M/50	Cervical discectomy and fusion	POD 7	Weakness of limbs	Hyporeflexia	No	VII, IX, X	No	Yes	Missing^b^	Inexcitable	Missing^b^	IVIg	No	G5/G2	22 mo
14	Huang, 2015 (11)	M/53	Cervical laminoplasty	POD 3	Weakness of limbs	Hyporeflexia	No	III, IX, X	No	Yes	Missing^b^	Axonal form	Yes	IVIg	No	G5/G4	22 mo
15	Huang, 2015 (11)	M/69	Lumbar interbody fusion	POD 2	Weakness of limbs	Hyporeflexia	No	IX, X	No	Yes	Missing^b^	Axonal form	Missing^b^	IVIg	No	G5/G5	11 mo
16	Huang, 2015 (11)	M/58	Cervical discectomy and fusion	POD 3	Weakness of limbs	Hyporeflexia	No	IX, X	No	No	Missing^b^	Demyelinating form	Yes	IVIg	No	G4/G1	7 mo
17	Hendawi, 2015 (24)	M/52	Pelvic fracture fixation	POD 14	Weakness and numbness of limbs	Areflexia	Numbness in limbs	No	No	Yes	Missing^b^	Missing^b^	Yes	IVIg, Steroids	No	G5/ improved^c^	Missing^b^
18	Al-Hashel, 2013 (25)	F/47	Internal fixation of tibial bones	POD 7	Weakness of limbs	Areflexia	No	VII, IX, X	No	Yes	Missing^b^	Demyelinating form	Missing^b^	IVIg	No	G5/G3	1 mo
19	Battaglia, 2013 (26)	F/73	Lumbar kyphoplasty	POD 14	Weakness of limbs	Areflexia	Hypoesthesia in limbs	VII, IX, X	No	No	Negative	Demyelinating form	Yes	IVIg	Intracranial hematoma	G4/G1	4 mo
20	Jakes, 2013 (27)	M/44	Renal transplantation	POD 3	Weakness of legs	Areflexia	No	No	No	No	Missing^b^	Axonal form	Yes	PE	No	G5/G0	2 mo
21	Cingoz, 2012 (28)	M/67	Coronary artery bypass surgery	POD 2	Weakness and paresthesia of legs	Areflexia	Paresthesia in legs	No	No	No	Missing^b^	Missing^b^	Missing^b^	PE	No	G4/G0	10 d
22	Miscusi, 2012 (29)	M/55	Chondroma resection	POD 2	Weakness and numbness of limbs	Areflexia	Numbness in limbs	No	No	No	GM1	Axonal form	Yes	IVIg	Cervical chondroma	G4/G4	36 mo
23	Heyworth, 2011 (30)	M/64	Hip arthroplasty	POD 16	Weakness and paresthesia of limbs	Areflexia	Paresthesia in limbs	VII	Hypertensive attacks	No	Missing^b^	Demyelinating form	Missing^b^	IVIg	No	G3/G2	12 mo
24	Cheng, 2011 (31)	F/59	Meningioma resection	POD 8	Weakness of legs	Areflexia	Hypoesthesia in limbs	III, VII, IX, X	No	No	Missing^b^	Axonal form	Yes	IVIg	Thoracic meningioma	G4/G4	7 mo
25	Son, 2011 (32)	M/50	Thoracic spine fusion	POD 10	Numbness of hands	Areflexia	Numbness in hands	VII	No	Yes	Missing^b^	Demyelinating form	Missing^b^	IVIg	No	G5/G1	2 mo
26	Algahtani, 2009 (33)	F/71	Coronary artery bypass surgery	POD 4	Weakness of legs	Areflexia	Pain in legs	No	No	Yes	Missing^b^	Axonal form	Both elevated	PE	No	G5/ Improved^c^	2.5 mo
27	Algahtani, 2009 (33)	M/77	Coronary artery bypass surgery	POD 3	Weakness of limbs	Areflexia	Numbness in limbs	No	No	No	Missing^b^	Demyelinating form	Yes	IVIg	No	G4/G4	1 mo
28	Lee, 2009 (34)	M/59	Pelvic fracture fixation	POD 9	Weakness of limbs	Areflexia	Numbness and tingling in limbs	No	No	No	Negative	Demyelinating form	Yes	PE	No	G4/G1	6 mo
29	Aluka, 2009 (35)	F/40	Bariatric surgery	POD 3	Numbness and tingling of legs	Areflexia	Hypoesthesia in limbs	No	No	No	Missing^b^	Axonal form	Normal	Supportive care	No	G3/G0	6 mo
30	Keithi-Reddy, 2007 (36)	M/48	Renal transplantation	POD 7	Numbness and tingling of legs	Areflexia	Numbness and tingling of legs	No	No	No	Missing^b^	Demyelinating form	Missing^b^	IVIg	Active CMV infection	G5/ Improved^c^	1 mo
31	Lin, 2006 (37)	F/22	Facial surgery	POD 3	Weakness of limbs	Areflexia	Numbness in legs	No	No	No	Missing^b^	Demyelinating form	Yes	Supportive care	No	G4/G0	1.5 mo
32	Falk, 2006 (38)	M/58	Lung transplantation	POD 12	Weakness of limbs	Areflexia	No	III, IV, VI	No	No	Missing^b^	Axonal form	Normal	PE	No	G4/G1	18 m
33	Rodriguez, 2002 (39)	M/18	Stem cell transplantation	POD 2	Paresthesia of limbs	Missing^b^	Paresthesia in limbs	No	No	Yes	Missing^b^	Axonal form	Yes	IVIg	T-cell lymphoma	G6/G6	3 mo
34	Koc, 2002 (40)	M/68	Hemicolectomy	POD 4	Paresthesia of limbs	Missing^b^	Paresthesia in limbs	IX, X	No	Yes	Missing^b^	Demyelinating form	Missing^b^	IVIg	Intestinal ad-enocarcinoma	G5/G0	12 mo

EMG, Electromyogram; AD, Albuminocytological dissociation; CSF, Cerebrospinal fluid; M, male; F, female; POD, Postoperative day; h, hour; d, day; mo, month; y, year; IVIg, Intravenous Immunoglobulin; PE, Plasma exchange; CMV, Cytomegalovirus; *C. jejuni*, *Campylobacter jejuni*; BOD, days before onset; MFS, Miller–Fisher syndrome. ^a^Grading system (41): 0, A healthy state; 1, Minor symptoms and capable of running; 2, Able to walk 10 m or more without assistance but unable to run; 3, Able to walk 10 m across an open space with help; 4, Bedridden or chair-bound; 5, Requiring assisted ventilation for at least part of the day; 6, Dead. ^b^Missing: Clinical data were lacking for the patient. ^c^Improved: The symptoms were improved, however, it was difficult to assess the disease severity, as clinical data were lacking for the patient.

## Retrieval strategy

The studies cited in this review were retrieved through an electronic search of the MEDLINE/PubMed and Embase datasets. The following keyword combinations were used to preliminarily select the articles to be evaluated: (“Guillain-Barre syndrome” or “GBS” or “acute inflammatory demyelinating polyneuropathy” or “AIDP” or “acute motor axonal neuropathy” or “AMAN” or “acute motor sensory axonalneuropathy” or “AMSAN”) AND (“surgery” or “operation” or “post-operative” or “post-surgical”). Most of the elected studies were published between 2002 and 2022. Only papers in English were reviewed. Articles were selected for their relevance, with a preference for new papers. Some other relevant papers known by the authors were also included.

## Epidemiology

Post-surgical GBS is a rare neurological disease. Its exact incidence is unclear, because rates reported in the literature vary due to differences in the numbers of patients studied. For example, in one case series of 63 GBS patients, 6 cases (9.5%) were diagnosed with GBS within 6 weeks after surgery (8). This indicated that the relative risk of developing GBS was 13.1 times higher than the incidence in the study population, which translated to an incidence of GBS of 4.5 per 100,000 surgeries. In a study of 69 GBS patients, 4 cases (5.8%) developed GBS within 6 weeks of surgery (7). This means that the relative risk of developing GBS was 6 times higher than the incidence in the general population, which translated to an incidence of 6.28 per 100,000 operations. In a study of 36 GBS patients, 7 patients (19.4%) developed GBS following surgery (10), which was a greater percentage than that observed in the two aforementioned studies (7, 8). Finally, in a 2015 retrospective study of spinal surgery patients, Huang and colleagues observed that the incidence of GBS after surgery was up to 51.5 per 100,000 surgeries (11). Although the incidences of post-surgical GBS varied widely in these studies, they indicate that the risk of developing GBS after surgery is significantly increased. Although surgery may increase the incidence of GBS, the pathological process is still unclear.

## Subtypes of GBS and post-surgical GBS

The most common subtypes of GBS are the demyelinating form (i.e., acute inflammatory demyelinating polyneuropathy); the axonal form (i.e., acute motor axonal neuropathy, acute motor-sensory axonal neuropathy); and its variant form [i.e., Miller-Fisher syndrome (MFS)]. All three forms of GBS have been reported to occur following surgery (see Table 1). However, differentiation between the three GBS subtypes is not easy during the early stage of the disease. Although distinguishing the three subtypes has no diagnostic value, it is important to do so because each has a different prognosis and treatment strategy.

The demyelinating form is a sensorimotor subtype of GBS, which is characterized primarily by paresis and mild sensory dysfunction. The axonal form is characterized by more rapid paresis, presence of more frequent cranial nerve deficits, and autonomic dysfunction. MFS is characterized by ophthalmoplegia, areflexia, and ataxia. Classification into demyelinating or axonal forms of GBS is mainly based on electrophysiological studies and subsequently supported by the presence or absence of specific anti-ganglioside antibodies (Tables 2, 3). With regard to post-surgical GBS, the axonal subtype is more common than the demyelinating subtype (10, 43, 44). This is consistent with the study of Staff and colleagues, who observed that nerve biopsies from 21 patients with post-surgical neuropathies showed more axonal degeneration than segmental demyelination (43). Of the 34 patients analyzed in the present study, 14 had the demyelinating subtype of GBS, 14 had the axonal subtype, and 1 had MFS. In the remaining 4 patients, however, it was difficult to determine which GBS subtype they had, as clinical data were lacking for these patients. Since the subtype in 5 of the 34 patients (14.7%) was indeterminable, it may bias somewhat the subtype analysis.

**Table 2:** Clinical characteristics of post-surgical Guillain-Barré syndrome and its subtypes.

Clinical features of typical post-surgical GBS
Unexplainable, progressive, symmetrical weakness in limbs (sometimes starting only in legs)
Hyporeflexia or areflexia in weak limbs
Mild or moderate sensory manifestations or signs (pain is often present)
Cranial nerve involvement (especially bulbar weakness, facial weakness, ocular anomalies)
Respiratory muscle involvement
Elevated protein concentration in CSF
Typical electrophysiological features (see Table 3)
Demyelinating form (AIDP)
Paresis primarily, mild sensory dysfunction
Neurophysiological criteria for demyelinating GBS (see Table 3)
Axonal form (AMAN and AMSAN)
Rapid-onset paresis, with or without sensory dysfunction; respiratory dysfunction
Anti-ganglioside antibodies against GM1, GM1b, GD1a, and GalNAc-GD1a
Neurophysiological criteria for axonal GBS (see Table 3)
Miller-Fisher syndrome
Opthalmoplegia, ataxia, areflexia
Anti-ganglioside antibodies against GD3, GT1a, and GQ1b

AIDP, acute inflammatory demyelinating polyradiculoneuropathy; AMAN, acute motor axonal neuropathy; AMSAN, Acute motor-sensory axonal neuropathy; CSF, cerebrospinal fluid; GBS, Guillain-Barré syndrome.

**Table 3:** Neurophysiological criteria for Guillain-Barré syndrome.^a^

Demyelinating form (AIDP)
At least one of the features mentioned below in at least two nerves, or at least two of the features mentioned below in one nerve if all other nerves are inexcitable and dCMAP >10% LLN
Motor conduction velocity < 90% LLN (85%, if dCMAP <50% LLN)
Distal motor latency >110% ULN (>120%, if dCMAP <100% LLN)
pCMAP/dCMAP ratio < 50% and dCMAP >20% LLN
F-wave latency >120% ULN
Axonal form (AMAN and AMSAN)
No features of AIDP or demyelination found in one nerve if dCMAP <10% LLN
Inexcitable
No measurable dCMAP in all nerves or dCMAP measured in only one nerve with dCMAP <10% LLN

AIDP, acute inflammatory demyelinating polyradiculoneuropathy; AMAN, acute motor axonal neuropathy; AMSAN, Acute motor-sensory axonal neuropathy; dCMAP, compound muscle action potential amplitude after distal stimulation; pCMAP, compound muscle action potential amplitude after proximal stimulation; LLN, lower limit of normal; ULN, upper limit of normal. ^a^See Uncini et al. for a review (42).

## Pathogenesis

Although more and more cases of post-surgical GBS have been documented after various types of surgical procedures, its underlying mechanism remains unclear. As the occurrence of GBS is either spatially or temporally separated from the operation, or both, a disruption of the inflammatory response or an autoimmune response is hypothesized to play a role in post-surgical GBS (43, 44). This is consistent with the finding that some patients with post-surgical GBS have elevated serum anti-ganglioside antibodies (20, 29), in the absence of viral or bacterial infection in their medical history. Also, nerve biopsies of 21 patients with non-traumatic post-surgical neuropathy showed endoneurial macrophage (CD-68 positive) infiltration and increased epineurial perivascular lymphocytic (CD45^+^ positive cells) inflammation of the nerves (43), suggesting that an immune response might play a role in surgery-associated neuropathies. However, in post-surgical GBS cases published over the past two decades, 7 of 34 patients developed neuropathy within 3 days after surgery (11, 14, 15, 17, 28, 29, 39). This three-day time interval between surgery and GBS onset is shorter than the expected time needed for an immune response to develop.

Thus, the simplest hypothesis for the GBS-surgery association could be that a nonspecific mechanism is responsible. In all likelihood, then, neuropathy may develop as a result of a complex interaction between various factors, such as a stress response, the surgical procedure itself, the primary comorbid disease, anesthesia, subclinical exogenous infections, and genetic factors. All of these factors, or a combination of them, can induce alterations in immune tolerance, predisposing T-cells to become dysregulated (43, 45–47). Although what triggers the immune attack on nerves after surgery is yet to be elucidated, it is evident from these studies that an immune-mediated mechanism may play an important role in patients with post-surgical GBS.

## Diagnosis

As with any patient with GBS, patients with surgery-related GBS are diagnosed largely based on clinical features (48, 49). In the surgical setting and considering the patient’s comorbidities, establishing a definitive diagnosis remains challenging because GBS symptoms significantly overlap with those resulting from other complications after surgery. Consistent with the European Academy of Neurology/Peripheral Nerve Society diagnostic criteria for GBS, the typical symptoms of post-surgical GBS are progressive, relatively symmetric bilateral muscle weakness in the extremities, and the presence of hyporeflexia or areflexia in the weak limbs at a minimum (50). In addition, some patients may present with mild to moderate peripheral sensory symptoms (e.g., numbness, paresthesia, pain).

When a patient exhibits unexplainable progressive muscle weakness or sensory symptoms in the limbs after surgery, GBS should be considered. Usually, weakness starts in the distal lower extremities, but, in some cases, it can start in the hands. Cranial nerve deficits may also be present, resulting in swallowing difficulties, facial weakness, or ocular anomalies. Weakness in the respiratory muscles can lead to the need for mechanical ventilation and prolonged weaning failure after surgery. Clinicians needs to bear in mind, however, that the clinical presentation of GBS can vary in regard to the involvement and severity of weakness, sensory symptoms, cranial nerve deficits, and respiratory dysfunction, which make it more difficult to diagnose. The typical clinical features of post-surgical GBS are summarized in Table 2.

Additional tests and examinations may be helpful to confirm the diagnosis or to exclude the possibility that other complications might be caused by something else. In GBS patients, cerebrospinal fluid (CSF) examination typically reveals albuminocytologic dissociation in the second week after symptom onset. Electromyography is also a mainstay of clinical investigations and is helpful for determining whether the disease is an axonal or demyelinating neuropathy.

## Clinical characteristics and disease course

To better appreciate the clinical course of post-surgical GBS, we compiled and analyzed the clinical data of cases published over the past 20 year. In total, we identified 34 patients, who met the diagnostic criteria of GBS and developed a peripheral neuropathy within 6 weeks of a surgical procedure and outside of the immediate post-operative period (<6 weeks and > 1 day before first symptoms) (8). These 34 patients comprise an exhaustive list of all post-surgical GBS patients reported in the literature from 2002 to 2022 (11, 13–40). Details of the clinical presentation in these patients are provided in Table 1. Analysis of these cases revealed that a wide spectrum of different surgical procedures can precede the onset of GBS, including spinal, cranial, cardiac, orthopedic, abdominal, and transplant surgery. The median time interval between surgical procedure and onset of neuropathy was 6.6 days (range, 1–17 days). The mean age of patients was 53.6 years (range, 18–87). The male-to-female gender ratio was 1.8 (22 men and 12 women).

All patients reported an acute or subacute onset of symptoms. Rapid progressive weakness of the limbs was the main clinical feature. The initial symptoms were limb weakness, numbness, paresthesia, or a combination of these symptoms. These symptoms could initially occur distally, but eventually they spread proximally. The bulbar nerves were often affected; the facial and ocular motor nerves were less often affected. Respiratory insufficiency occurred in 17 of the 34 cases (50.0%), requiring artificial ventilation. Sensory involvement was present in 23 patients (67.6%), which was described as paresthesia, hypoesthesia, hyperesthesia, numbness, and pain. Autonomic dysfunction occurred in 11.8% of the cases, sometimes as the first symptom. It led to cardiac arrhythmia, urine retention, hypertension, and gastric pain. Neuropathy severity was quantified at the nadir of symptoms and signs using the GBS disability score; the median score was 4.5 (range, 6–3), indicating confinement to bed or chair. Following intravenous immunoglobulin (IVIg), or plasma exchange (PE) treatment, or both, most patients experienced clinical improvement, albeit in different degrees; after these treatments, the median GBS disability score was 2.0 (range, 5–0). Despite timely immunotherapy, of the 34 patients, 11 (32.4%) were unable to walk without assistance, and 4 died (11.8%). Clearly, post-surgical GBS remains a severe disease, and better treatment is needed for some patients.

## Assessment scales

Patients with post-surgical GBS have highly diverse clinical manifestations and courses. Thus, assessment scales would be useful not only to detect GBS but also to monitor its progression. In this regard, the Peripheral Neuropathy Measures Outcome Study investigated a variety of assessment scales, with the goal of identifying an instrument capable of detecting GBS early in its course, reliably assessing disease severity, and capturing subtle disease-associated clinical changes. In that study, the research group reached consensus recommendations for the GBS Disability Scale and the Rasch-built Overall Disability Scale to measure disability, and the Medical Research Council (MRC) sum score and the new Rasch-built MRC score to measure muscle strength (51).

The GBS Disability Scale assesses functional status; its scores range from 0 (healthy) to 6 (dead) (41). The Rasch-built Overall Disability Scale assesses GBS patients on 24 items, grading each from 0 (unable to perform) to 2 (able to perform without difficulty) (52). The MRC sum score is a summation of MRC grades (range, 0–5) for performance of six pairs of muscles of the upper and lower limbs. The sum score ranges from 60 (normal strength) to 0 (total paralysis) (53). The Rasch-built MRC Scale assesses GBS patients on 12 items, grading each from 3 (normal strength) to 0 (paralysis) (54).

## Additional clinical investigations for GBS

### Lumbar puncture

A main feature of GBS is albuminocytological dissociation. This condition is defined by as an elevated CSF protein level but normal white blood cell count in the CSF. Of the 21 cases in the present analysis that had CSF data, 17 (80.9%) had albuminocytological dissociation. CSF analysis was not performed in 13 cases because a postoperative back wound precluded a spinal tap, or the patients declined the procedure.

CSF analysis is not necessary for diagnosing GBS. It is often performed in patients suspected of having GBS and is primarily performed to rule out other causes of muscle weakness rather than to confirm the GBS diagnosis. In the first week after onset of weakness, albuminocytologic dissociation occurred in approximately 50% of patients, although this percentage increased to no more than 80% in the third week (55). If CSF protein levels are normal, the diagnosis of GBS cannot be excluded, and repeat lumbar punctures are usually not recommended. Some patients with GBS or patients receiving IVIg exhibit a mild increase in CSF cell counts. Other differential diagnoses should be taken into account when the CSF cell count is more than 50 cells per microliter (3).

### Electrophysiological examination

Electrophysiological analyses play an important role in the diagnosis of GBS and discriminating between axonal and demyelinating subtypes. It is sometimes difficult to establish the proper diagnosis of GBS and the correct classification of GBS subtypes in the early phase of the disease, especially in atypical cases. Electrophysiological analyses can confirm subclinical abnormalities in peripheral neuropathy and aid the clinical diagnosis. To avoid the pitfalls due to reversible conduction failure and length-dependent reduction of compound muscle action potential amplitude, serial electrophysiological studies can help distinguish the GBS subtypes (56).

To acquire sufficient information, the electrophysiological examination usually includes nerve conduction studies (NCS) involving multisite stimulation. Records of F-waves and H-reflexes should be acquired in at least four motor nerves and three sensory nerves (5, 57). NCS abnormalities tend to be remarkable as the disease progresses, peaking 2 weeks after onset of muscle weakness (42). Although this examination can be performed at earlier stages of the disease course, NCS findings might still be normal, and F-waves might have prolonged latency or reduced persistence at this stage.

The electrophysiological characteristics of the affected nerves depend on the GBS subtype (3, 5). The nerves of patients with the demyelinating subtype exhibit prolonged distal motor latency, reduced nerve conduction velocity, prolonged F-wave latency, increased temporal dispersion, and conduction block. The nerves of patients with the axonal subtype exhibit decreased motor and/or sensory nerve amplitudes. Clinicians should pay attention to patients with reversible nerve conduction failure caused by impaired conduction at the node of Ranvier, as these patients are often misdiagnosed as having demyelinating instead of axonal GBS. The nerves of patients with MFS exhibit abnormal sensory conduction. Evoked sensory nerve amplitudes initially decrease and then return to normal with clinical improvement. Serial NCS might be helpful to reliably classify these GBS subtypes (58, 59).

### Testing for serum anti-ganglioside antibodies

Gangliosides are important cell membrane components in peripheral nerves. Serum anti-ganglioside antibodies have been found in patients exposed to *Campylobacter jejuni* and *Mycoplasma pneumoniae*, as well as in patients not exposed to these pathogens. Interestingly, most of these antibodies are associated with a specific subtype of GBS (3, 60). Testing for these antibodies can be helpful in the diagnosis and sub-classification of GBS. IgG autoantibodies to GM1, GM1b, GD1a, and GalNac-GD1a are frequently found in patients with acute motor axonal neuropathy or its axonal GBS variants, whereas IgG autoantibodies to GD3, GT1a, and GQ1b are often found in ophthalmoplegia and MFS. In contrast, an association between serum auto-antibodies and the demyelinating form of GBS has not been clearly established (61). Conclusions of axonal GBS or MFS based on anti-ganglioside antibodies should be made cautiously, however, because these tests have limited negative or positive predictive value (3). Although anti-ganglioside antibodies participate in the pathogenesis of GBS, their roles in accurately diagnosing GBS have not been confirmed. More research in this area is needed.

## Differential diagnosis

Due to varying degrees of dysfunction and different distribution of deficits in sensory and motor nerves, the clinical presentation of GBS varies. Also, numerous perioperative complications, such as infection, herniated disk, spinal cord infarct, metabolic dysfunction, etc., could mimic GBS symptoms. Sometimes, post-surgical GBS can be difficult to diagnose, especially in patients with atypical symptoms. Differential diagnosis of GBS depends on the clinician recognizing the localization of lesions in the nervous system (muscles, neuromuscular junction, peripheral nerves, spinal cord, brainstem, or conus lesion). Laboratory investigations (e.g., to identify electrolyte disturbances), CSF examination, spinal cord MRI, and electrophysiological examination are recommended to identify other causes of GBS-like symptoms. The presence of distal sensory nerve deficits supports a GBS diagnosis. However, if no sensory involvement exists, differential diagnoses such as myasthenia gravis, acute myopathy, or electrolyte disturbance should be excluded. If paralysis develops abruptly after spine surgery, an MRI of the spine should be performed and spinal cord compression should be ruled out.

The existence of post-surgical GBS highlights the importance of considering critical illness polyneuropathy (CIN) and critical illness myopathy (CIM) in the differential diagnosis. In patients with CIN and CIM, cranial nerve involvement leading to extraocular motor dysfunction, bifacial weakness, or swallowing difficulties are uncommon (62–64). The degree of sensory nerve involvement tends to be mild in CIN and normal in CIM. Albuminocytological dissociation in CSF and anti-ganglioside antibodies in serum are almost never observed in CIN and CIM. Myopathic motor unit potentials on needle electromyography can be found in CIM. Finally, CIN and CIM do not generally respond to IVIg and/or PE. Despite its clinical features, in a critical illness setting, it remains a diagnostic challenge to diagnose GBS early in the course of disease. Table 4 summarizes the characteristics of post-surgical GBS, critical illness polyneuropathy, critical illness myopathy, and GBS-like symptoms.

**Table 4:** Differentiating post-surgical GBS from CIP, CIM and GBS-like symptoms.^a^

	Post-surgical GBS	CIP and CIM	GBS-like symptoms
History	Operation	Critical illness, sepsis, systemic inflammatory response syndrome	The mechanical forces during and after the operation (transection, stretching, contusion, or compression); postoperative hematoma compression, electrolyte disturbance, etc.
Onset time	Usually several days after surgery and before ICU admission	Usually during ICU admission	Often immediately after these triggering factors
Clinical symptoms	Primarily symmetrical paresis with or without sensory dysfunction	Often symmetrical weakness, with mild sensory involvement in CIP and normal sensation in CIM	Often asymmetrical, compliant with involved nerves caused by mechanical forces
Autonomic dysfunction	Often	Less common	Often, especially when electrolyte disturbances occur
Cranial nerve deficits	Often	Almost never	Almost never
Serum anti-ganglioside antibodies	Elevated	Normal	Normal
CSF	Albuminocytologic dissociation	Normal	Normal
EMG	Axonal form: Reduction in CMAPs and SNAPs amplitude, normal or mildly reduced NCV, neuropathic MUAPs Demyelinating form: normal or mildly reduced in CMAPs and SNAPs amplitude, prolonged CMAP duration, normal NCV, normal MUAPs	CIP: Reduction in CMAPs and SNAPs amplitude, normal or mildly reduced NCV, neuropathic MUAPs CIM: Reduction in CMAPs amplitude, normal SNAPs, normal NCV, myopathic MUAPs	Compliant with involved nerves
Nerve biopsy	Polyneuropathy with inflammation	Axonal polyneuropathy without inflammation	—
Response to IVIg/PE	Good	No	No
Prognosis	Mostly no or mild deficits	Often persisting deficits	Improves after removal of mechanical forces

GBS, Guillain-Barré syndrome; CIP, critical illness polyneuropathy; CIM, critical illness myopathy; CMAP, compound muscle action potential; SNAP, sensory nerve action potential; NCV, nerve conduction velocity; MUAP, motor unit action potential; IVIg, intravenous Immunoglobulin; PE, Plasmapheresis; CSF, Cerebrospinal fluid. ^a^For a review of clinical characteristics, see (1–4).

## Treatment

Post-surgical GBS is a severe neurologic complication after surgery. Once the diagnosis is established, treatment should be administered as soon as possible before irreversible nerve injury occurs. Timely hospitalization is recommended. Treatment of post-surgical GBS is similar to that of non-surgical-associated GBS and usually involves general care and immunotherapy.

### General care

Even with adequate treatment, up to 5% of GBS patients die from complications. Until their condition begins to stabilize, all GBS patients should be hospitalized for observation. Multidisciplinary care is needed to prevent and treat serious complications. Respiratory insufficiency is more likely to occur in cases with rapid disease progression, facial and/or bulbar palsy, or severe weakness at hospital admission (64, 65). In the present study, 17 of the 34 cases (50.0%) with post-surgical GBS experienced respiratory insufficiency. Thus, clinicians need to pay attention to patients’ pulmonary function. In general, predictors of ventilator support include hypercarbia, hypoxemia, and a vital capacity of less than 15 mL/kg (66).

Although autonomic dysfunction occurs throughout the course of GBS, it mainly occurs during the progressive phase of the disease. Autonomic instability, such as cardiac arrhythmia, blood pressure instability, and gastrointestinal dysfunction, have been reported in the literature (18, 19, 23, 30), and it is sometimes a serious problem that can lead to sudden death. Thus, all patients at high risk for respiratory failure or autonomic dysfunction should be carefully monitored and transferred to an intensive care unit as soon as possible. Among the cohort of 34 cases we analyzed, 10 (29.4%) experienced choking while drinking, indicating that swallowing needs to be assessed early on so that patients at high risk of aspiration pneumonia can be identified and monitored. During the progressive phase, irritating sensory nerve symptoms described as paresthesia, numbness, pain, and tingling occurred in 16 of the 34 post-surgical GBS patients (47.1%). Early recognition of these symptoms is essential. Treatment with opioids, gabapentin, or carbamazepine improve sensory symptoms, but glucocorticoids have not (67). Other possible complications, such as urinary retention and deep-vein thrombosis, require careful management.

### Immunotherapy

Starting immunotherapy early on speeds recovery and improves prognosis. Although a definitive diagnosis of GBS is difficult early in the disease course, empirical treatment with IVIg or PE should be initiated, if GBS is suspected and sufficient evidence exists to support its tentative diagnosis.

PE therapy improves symptoms when given during the first 4 weeks after disease onset (68). Its effects, however, tends to be largest when treatment is applied within the first 2 weeks of symptom onset. PE is thought to nonspecifically remove circulating antibodies and complement factors. The usual empirical regimen is a total exchange of five plasma volumes over a period of 2 weeks (68). Among patients who are still able to walk, however, two plasma exchange sessions aids in amelioration of motor deficits more rapidly than does no PE.

Another form of immunotherapy given to GBS patients is IVIg. IVIg is given at a dose of 0.4 g/kg daily for 5 consecutive days (or 1 g/kg daily for 2 days) (69). IVIg is thought to neutralize neurotoxic antibodies and inhibit the autoantibody-mediated complement cascade. Because of large variations in the pharmacokinetics of IVIg across individuals, patients who have a smaller increase in serum IgG level after IVIg administration might have a poor outcome. There is no evidence to support a second course of IVIg is beneficial for patients who have a poor prognosis, or who continue to deteriorate, the International Guillain-Barré Syndrome Outcome Study indicates the need for treatment trials with other immune modulators in patients severely affected by GBS (70).

Since results from a randomized controlled trial (69) showed that IVIg is as effective as PE in patients who are unable to walk independently, IVIg has replaced PE as the preferred choice of treatment for GBS patients in many hospitals because of its minor adverse effects and greater convenience. However, patients with acute motor axonal neuropathy might have a better outcome when treated with PE than IVIg (71). The administration of PE followed by a course of IVIg does not confer significantly better therapeutic results than application of either PE or IVIg alone. After correcting for known prognostic factors, van Koningsveld et al. observed that treatment composed of methylprednisolone and IVIg conferred short-term therapeutic benefits for some GBS patients (72). However, overall this combination therapy did not result in significantly better outcomes than IVIg alone. Moreover, prednisolone or methylprednisolone failed to significantly improve long-term outcome (73).

## Prognosis

Although post-surgical GBS is potentially reversible, even when treated with standard therapy, patient outcome varies substantially. If left untreated, patients with post-surgical inflammatory neuropathy can either improve spontaneously or deteriorate (43). Recovery is associated with multiple factors (74–77), such as age, symptoms at nadir, type of therapy, primary disease, etc. In adults, severity of disease at nadir, such as being bedbound or requiring ventilation support, is usually considered to be a poor prognostic factor (78). Patients who can still walk 2 weeks after onset are likely to improve with or without treatment, but they may be left with some permanent neurologic impairment (79–81). Most improvement occurs during the first year after onset, but patients can recover further after this period. Even if they experience good functional recovery, many patients who survive GBS have to change their lifestyle (82).

## Conclusion

Post-surgical GBS is a rare but serious neurologic complication after surgery that can mimic various GBS-like symptoms. Its characteristic symptoms are rapidly progressive muscle weakness and areflexia, often accompanied by dysphagia, facial paralysis, or respiratory failure. When there is unexplained symmetrical weakness after a major surgical procedure, GBS should be considered. Early diagnosis and timely therapy are imperative to prevent further neurological damage. Electrophysiological examination of peripheral nerves and CSF analysis can aid the clinical diagnosis. Treatment with IVIg or PE can improve symptoms.

## Author contributions

XL: Formal analysis, Funding acquisition, Investigation, Writing – original draft, Writing – review & editing. CZ: Conceptualization, Supervision, Writing – review & editing.
